# Cytokines and Effector/Regulatory Cells Characterization in the Physiopathology of Cutaneous Lupus Erythematous: A Cross-Sectional Study

**DOI:** 10.1155/2016/7074829

**Published:** 2016-03-06

**Authors:** Silvia Méndez-Flores, Gabriela Hernández-Molina, Ana Bety Enríquez, David Faz-Muñoz, Yeraldin Esquivel, Carlos Pacheco-Molina, Janette Furuzawa-Carballeda

**Affiliations:** ^1^Department of Dermatology, Instituto Nacional de Ciencias Médicas y Nutrición, Vasco de Quiroga No. 15, Colonia Belisario Dominguez Sección XVI, 14080 Mexico City, DF, Mexico; ^2^Department of Immunology and Rheumatology, Instituto Nacional de Ciencias Médicas y Nutrición, Vasco de Quiroga No. 15, Colonia Belisario Dominguez Sección XVI, 14080 Mexico City, DF, Mexico

## Abstract

We compared the presence of diverse cytokines and regulatory T and B cells in skin biopsies of discoid lupus erythematosus (DLE) and subacute cutaneous lupus erythematosus (SCLE). We included 19 patients with DLE, 13 with SCLE, 8 healthy controls, and 5 patients with hypertrophic scars. We assessed the CLASI activity score. To determine IL-22-producing cells and the subpopulation of CD4^+^/IL-17A^+^-, CD4^+^/IL-4^+^-, and CD4^+^/IFN-*γ* ^+^-expressing T cells, CD123^+^/IDO^+^ pDCs, CD25^+^/Foxp3^+^ Tregs, and CD20^+^/IL-10^+^-producing B cells, an immunostaining procedure was performed. Also intracellular IL-22, IL-17, IL-4, IFN-*γ*, and Foxp3 in CD4 T cells, IL-10 in B cells, and IDO in pDCs were analyzed by flow cytometry in peripheral blood. The main cellular participation in both lupus groups was IL-17- and IL-22-producing cell responses both at skin and at peripheral blood but prevailed in DLE. The CLASI activity scores negatively correlated with Th22 subpopulation and positively correlated with CD25^+^/Foxp3^+^ Treg cells. In conclusion a proinflammatory and regulatory imbalance coexists in cutaneous lupus, both responses being more intense in DLE.

## 1. Introduction

Cutaneous lupus erythematosus is a chronic autoimmune disease with a broad spectrum of cutaneous manifestations that may precede systemic lupus erythematosus (SLE), stay as the only lupus feature, or occur at any stage of the disease among patients with SLE [1]. Overall skin involvement occurs in 70% of SLE patients. Based on the analysis of skin biopsies, Gilliam proposed a classification system for CLE dividing in specific and nonspecific lesions. Specific lesions that correspond to 23% of skin involvement are characterized by the presence of interphase dermatitis, mucin deposition in superficial dermis, and predominantly perivascular and periadnexal infiltrates. CLE-specific lesions are further divided in acute (ACLE), subacute (SCLE), and chronic (CCLE) varieties; this last category includes the discoid lupus erythematosus (DLE) [2]. Thus cutaneous manifestations are heterogeneous and often represent a clinical challenge, which in turn reflects the complexity of the underlying pathogenic mechanisms. In this sense, diverse mechanisms have been implicated in the physiopathogenesis of cutaneous lupus erythematosus such as environmental factors (ultraviolet light exposure, drugs, etc.), keratinocyte apoptosis, genetic susceptibility, B cell hyperactivity, the interaction of the innate and cell-mediated immunity, cytokine and chemokine release, and uncontrolled and persistent effector T cell responses that can drive the onset of skin lesions [3].

Nevertheless few studies have assessed the complex cytokine network and regulatory cells in cutaneous lupus. The cytokine network may participate in the amplification, maintenance of autoimmune response, and the induction of tissue injury, but also in the tissue repair. For instance, the expression of mRNA for IL-6 in basal keratinocytes of patients with DLE and SCLE [4] as well of IL-5, IL-10, IL-2, IL-4, and IFN-*γ* in ACLE, SCLE, and DLE has been reported [5]. More recently, the participation of IL-17 [6] and decreased number of Treg cells, and in consequence the loss of tolerance, have also been recognized in cutaneous lupus [7].

Thus, this study evaluated the participation of circulating and skin IL-22-, IL-17-, IL-4-, and IFN-*γ*-producing cells, as well as regulatory subsets (Tregs, Bregs, and pDCregs) and serum levels of IL-22, IL-17, and IL-10 in patients with DLE and SCLE and with regard to disease activity.

## 2. Material and Methods

### 2.1. Patients

This was an exploratory, observational, and cross-sectional study conducted in a tertiary care center between February 2013 and December 2014. We included 35 patients with cutaneous lupus: 20 with DLE and 15 with SCLE. To be eligible, patients had to meet classification criteria for systemic lupus erythematosus according to the ACR criteria [8] and to have an active lupus specific lesion compatible with DLE or SCLE. The diagnosis of cutaneous lupus was established in consensus by a rheumatologist and a dermatologist, as well as biopsy proven. In addition, patients should not be under topical treatment including steroids within the last 4 weeks except for emollients. However, patients were allowed to maintain their basic systemic treatment such as oral steroids and immunosuppressors. Patients were excluded if they had any concomitant cutaneous lesion not attributed to lupus or an overlap autoimmune condition. We also included a total of 16 healthy donors (HD) matched by age ±5 years and 5 patients with hypertrophic scars (HSc). The controls did not have any autoimmune disease or concurrent infection or receive prednisone or immunosuppressive therapy.

Clinical activity was measured by the Cutaneous Lupus Erythematosus Disease Area and Severity Index (CLASI), a validated index to quantify disease severity [9]. As this instrument measures both activity and damage, for the present study we only used the activity domain that ranges from 0 to 70 (higher scores are indicative of more severity).

In addition, patients' clinical records were carefully reviewed according to a preestablished protocol to retrospectively collect demographics as well as clinical and serologic features.

### 2.2. Tissue, Peripheral Blood, and Plasma Samples

Skin punch biopsies (4 mm diameter) were performed, fixed in formalin, and evaluated with hematoxylin-eosin staining for the presence of classic histologic cutaneous lupus features. Then the rest of the specimen was stored for immunohistochemistry. Microscopic review was conducted in a blinded manner to the diagnosis by one independent observer (Janette Furuzawa-Carballeda).

Overall, most of the cutaneous lupus biopsies corresponded to photoexposed areas localized at thorax, arms, or scalp. Control tissue biopsies were also taken from photoexposed areas and when possible from the same anatomical region.

In addition, fifteen milliliters of venous blood from each subject was obtained by venipuncture in tubes with EDTA. Blood was centrifuged at 2000 rpm for 20 minutes. Plasma was isolated and stored at −70°C until use.

### 2.3. Standard Laboratory Assessments

The evaluation included erythrocyte sedimentation rate (ESR) determined by Westergren method, blood chemistry, anti-double stranded DNA (ds-DNA) antibodies determined by ELISA, and C3 and C4 quantified by ELISA.

### 2.4. Immunohistochemistry

IL-22-expressing cells were determined in 4 *µ*m thick sections of available formalin-fixed paraffin embedded tissue. Endogenous peroxidase and binding of nonspecific proteins were blocked with 3% H_2_O_2_ and 3% normal serum, respectively. Tissues were incubated with goat polyclonal antihuman IL-22 antibody (Santa Cruz Biotechnology, Santa Cruz, CA, USA) at 10 *µ*g/mL. Binding was identified with biotinylated donkey anti-goat IgG antibody (ABC Staining System; Santa Cruz Biotechnology). Slides were incubated with horseradish peroxidase- (HRP-) streptavidin, followed by incubation with the peroxidase substrate 3,3′-diaminobenzidine (DAB) (SIGMA-Aldrich) for 10 min. The sections were counterstained with hematoxylin, dehydrated with alcohol and xylene, and mounted in resin. Negative controls staining was performed with normal human serum diluted 1 : 100, instead of primary antibody, and the IHC universal negative control reagent specifically designed to work with rabbit, mouse, and goat antibodies (IHC universal negative control reagent, Enzo Life Sciences, Inc., Farmingdale, NY, USA, ADI-950-231). The reactive blank was incubated with phosphate buffer saline-egg albumin (SIGMA-Aldrich) instead of the primary antibody. Both controls excluded nonspecific staining or endogenous enzymatic activities [10].

### 2.5. Double-Staining Procedure

To determine the subpopulation of CD4^+^/IL-17A^+^-, CD4^+^/IL-4^+^-, and CD4^+^/IFN-*γ* ^+^-expressing T cells, CD25^+^/Foxp3^+^ regulatory T cells, CD20^+^/IL-10^+^-producing B cells, and CD123^+^/IDO^+^ pDC cells a simultaneous detection was performed [EnVision*™* G|2 Doublestain System (Dako, Glostrup, Denmark)]. The procedure is a sequential double staining where the first antigen [normal serum as negative control, rabbit polyclonal anti-IL-17A, anti-IL-4, anti-IFN-*γ*, and anti-IDO IgG antibody or mouse monoclonal anti-IL-10 or anti-Foxp3 IgG_1_ antibody (Santa Cruz Biotechnology) at 10 *µ*g/mL)] was visualized using horseradish peroxidase (HRP)/3,3′-diaminobenzidine (DAB) and the second antigen [normal serum as negative control or second primary rabbit polyclonal anti-CD20, anti-CD25 IgG antibody or mouse monoclonal anti-CD4, anti-IgG_1_ antibody (Santa Cruz Biotechnology) or anti-CD123 IgG antibody (Abcam pcl, CA, UK) at 10 *µ*g/mL] was visualized using alkaline phosphatase (AP)/Permanent Red. Tissues were counterstained with hematoxylin and mounted in aqueous mounting medium. Cytokine-expressing cells as well as double positive CD4^+^/IL-17A^+^-, CD4^+^/IL-4^+^-, and CD4^+^/IFN-*γ* ^+^-expressing T cells, CD25^+^/Foxp3^+^ regulatory T cells, CD20^+^/IL-10^+^-producing B cells, and CD123/IDO pDCs were assessed by estimating the number of positively staining cells in two fields (×320) and were reported as the percentage of immunoreactive cells of the inflammatory infiltrates located at epidermis and dermis. Results are expressed as the mean ± standard error of the mean (SEM) of cells quantified by the program Image Pro Plus version 5·1·1 [10].

### 2.6. Peripheral Blood Mononuclear Cell Isolation

Fifteen-milliliter sample of venous blood was obtained from each subject. Peripheral blood mononuclear cells (PBMCs) were obtained by gradient centrifugation on Lymphoprep (Axis-Shield PoC AS, Oslo, Norway).

### 2.7. Flow Cytometry

PBMCs were labeled with 5 *µ*L of antihuman CD3-FITC-labeled, antihuman CD4-PeCy5-labeled, and antihuman CD161-APC-conjugated monoclonal antibodies (BD Biosciences, San Jose, CA); antihuman CD3-FITC-labeled, antihuman CD4-PeCy5-labeled, and antihuman CD14-APC-conjugated monoclonal antibodies (BD Biosciences); antihuman CD19-APC-labeled, antihuman CD24-FITC-conjugated, and antihuman CD38-PeCy5-labeled monoclonal antibodies (BD Biosciences); or antihuman CCR6-PerCP/Cy5.5-conjugated and antihuman CD123-FITC-labeled monoclonal antibodies (BD Biosciences) in separated tubes during 20 min at 37°C in the dark. Cells were permeabilized with 200 *µ*L of cytofix/cytoperm solution (BD Biosciences) at 4°C for 30 min. Intracellular staining was performed with an antihuman IL-22-PE-, IL-17A-PE-, IL-4-PE-, IFN-*γ*-PE-, Foxp3-PE-, IL-10-PE-, and IDO-PE-labeled mouse monoclonal antibodies (BD Biosciences) for 30 min at 4°C in the dark. An electronic gate was made for CD3^+^/CD4^+^/CD161^−^ cells, CD3^+^/CD4^+^/CD161^+^ cells, CD3^+^/CD4^+^/CD14^−^ cells, CD3^+^/CD4^+^/CD25^hi^ cells, CD19^+^/CD38^hi^/CD24^hi^ cells, and CD123^hi^/CD196^+^ cells (Figure 5(h)). Results are expressed as the relative percentage of IL-22^+^, IL-17A^+^, IL-4^+^, IFN-*γ* ^+^, Foxp3^+^, IL-10^+^, and IDO^+^ expressing cells in each gate. As isotype control, IgG1-FITC/IgG1-PE/CD45-PeCy5 mouse IgG1* kappa* (BD Tritest, BD Biosciences) was used to set the threshold and gates in the cytometer. We ran an unstained (autofluorescence control) and permeabilized PBMCs sample. Autofluorescence control was compared to single stained cell positive controls to confirm that the stained cells were on scale for each parameter. Besides, BD CaliBRITE 3 beads were used to adjust instrument settings, set fluorescence compensation, and check instrument sensitivity (BD CaliBRITE, BD Biosciences). Fluorescence minus one (FMO) controls were stained in parallel using the panel of antibodies with sequential omission of one antibody, with the exception of the anti-IL-22, anti-IL-17A, anti-IL-4, anti-IFN-*γ*, anti-Foxp3, anti-IL-10, and anti-IDO antibody, which was replaced by an isotype control rather than simply omitted. Finally, T subsets were analyzed by flow cytometry with an Accuri C6 (BD Biosciences). A total of 500,000–1,000,000 events were recorded for each sample and analyzed with the FlowJo X software (Tree Star, Inc.) [11].

### 2.8. ELISA Assays

Serum levels of IL-22, IL-17A, and IL-10 were measured by enzyme-linked immunosorbent assays using commercial kits (BioLegend Inc., San Diego, CA, USA) according to instructions provided by the manufacturer.

### 2.9. Ethical Considerations

This work was performed according to the principles expressed in the Declaration of Helsinki. The study was approved by the ethical committee from the Instituto Nacional de Ciencias Médicas y Nutrición, and a written informed consent was obtained from all subjects.

### 2.10. Statistics

Descriptive statistic was performed and categorical variables were compared using the Chi-2 test or Fisher's exact test. One-way analysis of variance on ranks by Holm-Sidak method and Dunn's test was performed for all pairwise multiple comparison procedures. We reported nonparametric correlations using Spearman coefficients among the disease activity CLASI score and the immunohistochemistry and serological results.

Statistical analysis was done using the Sigma Stat 11.2 program (Aspire Software International, Leesburg, VA, USA). Data were expressed as the median, range, and mean ± standard deviation (SD)/standard error of the mean (SEM). The *P* values smaller than or equal to 0.05 were considered as significant. This study conforms to STROBE statement along with references to STROBE and the broader EQUATOR guidelines [12].

## 3. Results

### 3.1. Clinical and Demographic Characterization of Patients

**Figure 1:**
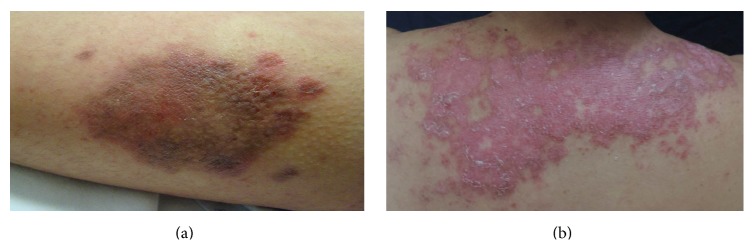
(a) Discoid lupus erythematosus. (b) Subacute lupus erythematosus.

We included a total of 35 patients with cutaneous lupus without other autoimmune comorbidity; 20 had DLE (Figure 1(a)) and 15 patients had SCLE (Figure 1(b)). Ninety four percent of cutaneous lupus patients and all the controls were women. Patient's demographic; laboratory; and clinical data are shown in Table 1. ESR levels, cutaneous activity score, anti-dsDNA antibody levels, dose of prednisone, and antimalarials were conspicuously higher in SCLE compared with DLE patients.

### 3.2. Histological Findings

**Figure 2:**
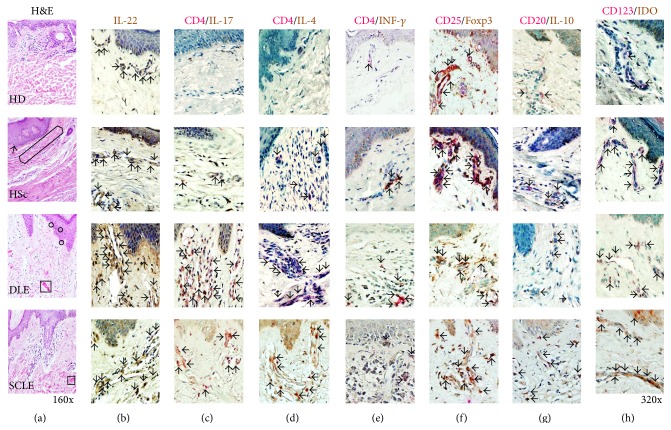
Tissue architecture, cytokines, CD4 effector T cells, and regulatory cells in cutaneous lupus erythematosus. (a) Representative photomicrograph from a HD (healthy donor), HSc (hypertrophic scar), DLE (discoid lupus erythematosus), and SCLE (subacute cutaneous lupus erythematosus) tissues stained by H&E technique. Original magnification was ×160. Immunohistochemistry for (b) IL-22, (c) CD4/IL-17A, (d) CD4/IL-4, (e) CD4/IFN-*γ*, (f) CD25/Foxp3, (g) CD20/IL-10, and (h) CD123/IDO cells. Arrows depict immunoreactive cells. Original magnification was ×320.

DLE tissue showed that dermis had perivascular and periadnexal lymphohistiocytic infiltrates under the interface dermatitis. A degeneration of the basal layer, apoptotic keratinocytes (circles in Figure 2), and a marked thickening of the basement membrane were observed. Moreover, deposition of dermal mucin was detected (square in Figure 2).

On the other hand, characteristic histopathologic alterations observed in SCLE included vacuolar alteration of the basal cell layer and subepidermal, periappendiceal, and/or perivascular inflammatory cell infiltrate. Epidermal changes, such as atrophy and mucin within the dermis, were detected (square in Figure 2).

Finally, HSc had flattening of epidermis with obliteration of the rete ridges and hyperkeratosis (arrow in Figure 2). The collagen was seen spanning full thickness above one-third of reticular dermis in nodules oriented parallel to each other (hexagon in Figure 2). HSc showed mild perivascular chronic inflammatory infiltrate end loss of cutaneous appendages (Figure 2).

### 3.3. Proinflammatory/Antifibrogenic Cytokine Expression and CD4 Effector T Cells in Tissue from Patients with Cutaneous Lupus

**Figure 3:**
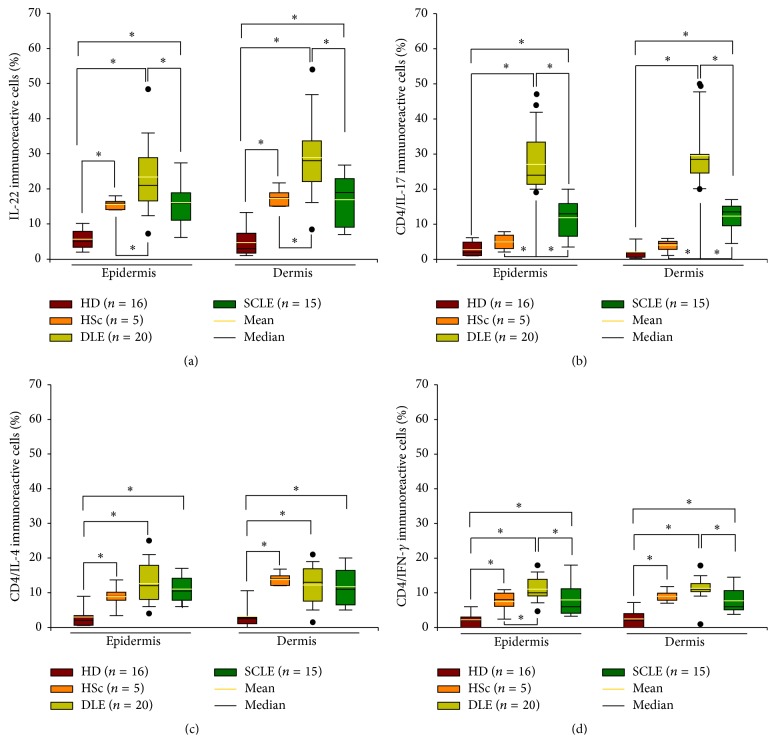
Cytokine expression and CD4 effector T cells in tissue from patients with cutaneous lupus. Immunohistochemistry for (a) IL-22^+^, (b) CD4^+^/IL-17^+^, (c) CD4^+^/IL-4^+^, and (d) CD4^+^/IFN-*γ* ^+^ T cells. Results are expressed as mean (yellow line), median (black line), and 5th/95th percentiles. HD: healthy donor, HSc: hypertrophic scar, DLE: discoid lupus erythematosus, and SCLE: subacute cutaneous lupus erythematosus. ^*∗*^ *P* < 0.05.

IL-22^+^ cell percentage was increased in dermis and epidermis of DLE tissue samples when compared to SCLE, HSc, and healthy control group (Figure 3(a), Table 2). However, IL-22^+^ cell number in SCLE only was statistically significant when compared to healthy donors (Figure 3(a), Table 2).

IL-17A/CD4 T cell frequency was conspicuously higher in dermis and epidermis of DLE and SCLE tissue patients versus HSc and HD tissues. Moreover, a higher immunoreactive cell number was found in tissue from DLE compared with SCLE patients (Figure 3(b), Table 2).

Tissue from DLE patients had significantly higher percentage of IFN-*γ*/CD4 T cells versus SCLE patients, HSc, and healthy donor control groups. Meanwhile, the number of IFN-*γ*/CD4 T cells in the skin from SCLE patients was increased when compared with healthy donor group (Figure 3(d), Table 2).

### 3.4. Anti-Inflammatory/Profibrogenic Cytokine Expression and CD4 Effector T Cells in Tissue from Patients with Cutaneous Lupus

Dermis and epidermis from DLE, SCLE, and HSc tissue patients had significantly higher IL-4/CD4 T cell percentage when compared to healthy donor group. There were no statistically significant differences amongst DLE and SCLE patient groups (Figure 3(c), Table 2).

### 3.5. Regulatory Cells in Tissue from Patients with Cutaneous Lupus

**Figure 4:**
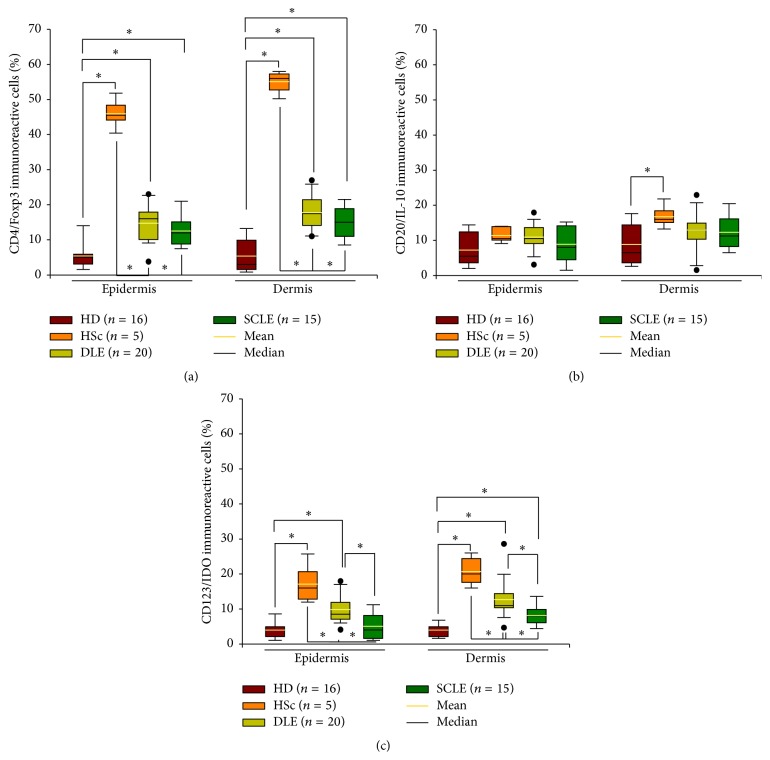
Regulatory cells in tissue from patients with cutaneous lupus. Immunohistochemistry for (a) CD4^+^/Foxp3^+^, (b) CD20^+^/IL-10^+^, and (c) CD123^+^/IDO^+^ cells. Results are expressed as mean (yellow line), median (black line), and 5th/95th percentiles. HD: healthy donor, HSc: hypertrophic scar, DLE: discoid lupus erythematosus, and SCLE: subacute cutaneous lupus erythematosus. ^*∗*^ *P* < 0.05.

Epidermis and dermis from HSc tissue showed a noticeably higher Treg cell frequency compared with tissue from healthy donors, DLE, and SCLE patients. Nonetheless, there were no statistical differences between tissue from DLE and SCLE patients (Figure 4(a), Table 2).

Only a slight increase of IL-10/CD20 B cell percentage was observed in dermis from HSc patients compared to healthy donors. Nonetheless, there were no differences amongst DLE, SCLE, and healthy donors (Figure 4(b) Table 2).

Cutaneous tissue from HSc patients had the highest percentage of CD123^+^/IDO^+^ cells. Further, statistically significant differences were found in DLE versus SCLE and healthy donor cutaneous tissue (Figure 4(c), Table 2).

### 3.6. Percentage of Circulating CD4^+^ T Cell Subpopulations

**Figure 5:**
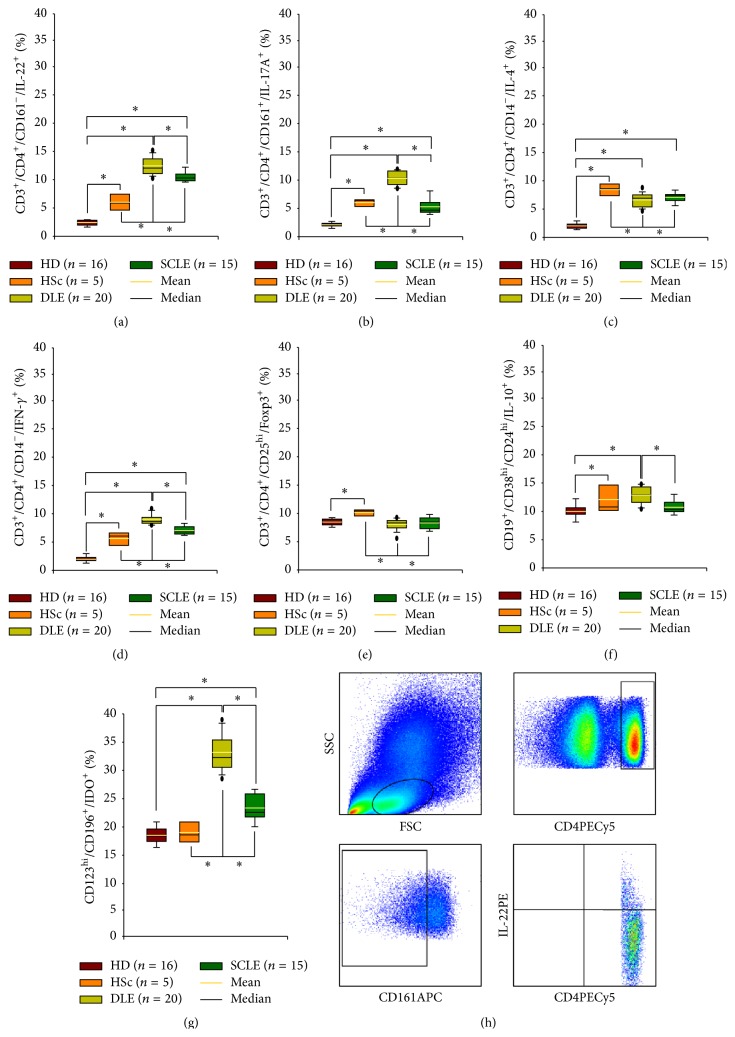
Circulating CD4 effector T and regulatory cells in patients with cutaneous lupus. (a) IL-22-producing CD4 T cells, (b) IL-17-secreting CD4 T cells, (c) IL-4-expressing CD4 T cells, (d) IFN-*γ*-producing CD4 T cells, (e) Foxp3-expressing CD4 T cells, (f) IL-10-producing B cells, and (g) IDO-expressing pDC cells. Results are expressed as mean (yellow line), median (black line), and 5th/95th percentiles. HD: healthy donor, HSc: hypertrophic scar, DLE: discoid lupus erythematosus, and SCLE: subacute cutaneous lupus erythematosus. (h) Representative flow plot. ^*∗*^ *P* < 0.05.

To determine the different T cell subpopulations, PBMCs were immunophenotyped and analyzed by flow cytometry. Relative percentage of proinflammatory circulating Th22, Th17, and Th1 cells from DLE patients was higher when compared with SCLE. Peripheral Th22, Th17, and Th1 subsets were increased in DLE and SCLE patients versus HSc patients and healthy donors. Moreover, there were a higher number of cells in DLE versus SCLE cutaneous tissue (Figures 5(a), 5(b), and 5(d), Table 3).

Regarding anti-inflammatory/profibrogenic Th2 cell percentage, there was a significant increase in HSc patients when compared with DLE and SCLE patients and healthy donors (Figure 5(c), Table 3). Peripheral IL-4-producing CD4 T cells were more abundant in DLE and SCLE patients versus healthy donors. Furthermore, there were no differences in Th2 relative percentage amongst SCLE and DLE patients (Figure 5(c), Table 3).

Finally, circulating regulatory T, B, and pDC cell percentage was evaluated. Forkhead box P3-expressing CD4 T cells showed a statistically significant increase only in tissue from HSc patients compared with skin from DLE and SCLE patients and healthy donors. There were no statistically significant differences between DLE and SCLE patients (Figure 5(e), Table 3).

Meanwhile, IL-10-producing B cell relative percentage was higher in DLE versus SCLE patients and healthy donors (Figure 5(f), Table 3). HSc patients had higher Breg cell percentage when compared with healthy donors (Figure 5(f), Table 3).

pDCreg cell percentage was conspicuously higher in DLE patients when compared with SCLE and HSc patients and healthy donors. Circulating pDCreg cells were increased in SCLE versus HSc and healthy donors (Figure 5(g), Table 3).

### 3.7. IL-22, IL-17A, and IL-10 Serum Levels

**Figure 6:**
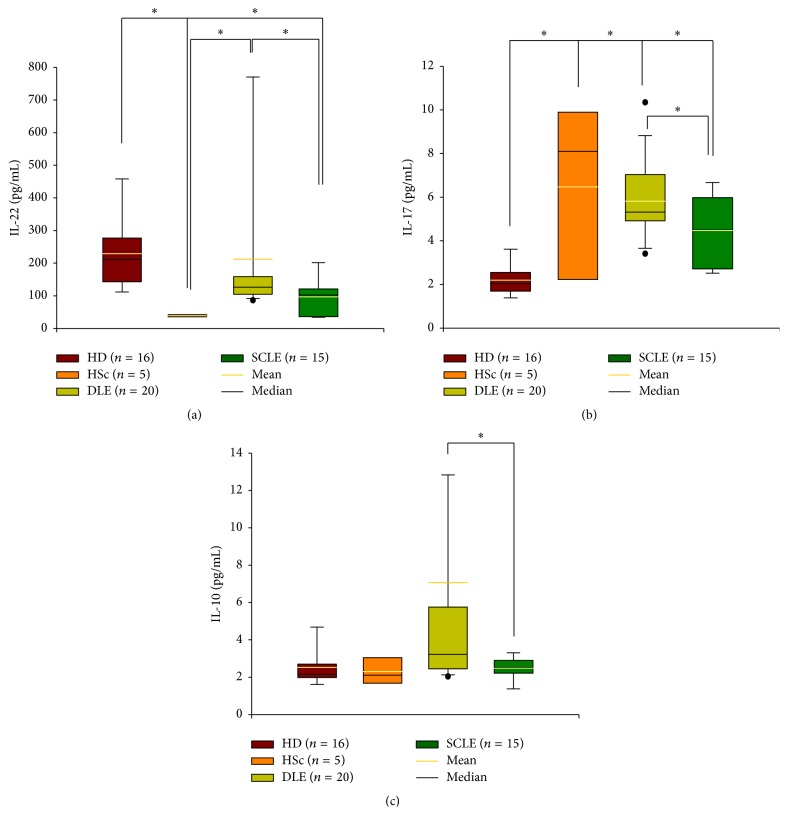
Cytokine levels in serum from patients with cutaneous lupus. (a) IL-22, (b) IL-17, and (c) IL-10 concentration. Results are expressed as mean (yellow line), median (black line), and 5th/95th percentiles. HD: healthy donor, HSc: hypertrophic scar, DLE: discoid lupus erythematosus, SCLE: subacute cutaneous lupus erythematosus.

Serum levels of IL-22, IL-17A, and IL-10 were determined in DLE, SCLE, healthy donors, and HSc by ELISA. IL-22 serum concentration in DLE patients (median: 126.4 pg/mL, range: 85.8–955.5 pg/mL) was higher when compared to SCLE patients (median: 101.9 pg/mL, range: 34.4–249.9 pg/mL; *P* = 0.05). In healthy donors the median serum concentration of IL-22 was 211.2 pg/mL (range: 106.3–517.5 pg/mL) and it was significantly higher in comparison with the SCLE and HSc patients (median: 37.5 pg/mL, range: 32.2–45.2 pg/mL) (Figure 6(a), Table 4).

In regard to IL-17 serum concentration, it was increased in DLE patients (median: 5.3 pg/mL, range: 3.4–10.4 pg/mL) versus SCLE patients (median: 4.5 pg/mL, range: 2.3–6.8 pg/mL; *P* = 0.05). IL-17 in healthy donors was significantly lower (median: 2.0 pg/mL, range: 1.1–3.9 pg/mL) when compared with DLE (*P* = 0.05) and SCLE and HSc patients (median: 8.1, range: 1.6–3.5; *P* = 0.05; Figure 6(b), Table 4).

DLE patients had higher levels of IL-10 in serum (median: 3.2 pg/mL, range: 2.0–51.9 pg/mL) when compared with SCLE patients (median: 2.5 pg/mL, range: 1.1–3.5 pg/mL; *P* = 0.05; Figure 6(c), Table 4). There were no significant differences in the serum concentrations among cutaneous lupus groups, healthy donors (median: 2.1 pg/mL, range: 1.6–5.4 pg/mL), and HSc (median: 2.1 pg/mL, range: 1.6–3.5 pg/mL) groups (Figure 6(c), Table 4).

### 3.8. Correlation in Cutaneous Lupus Erythematosus between Tissue Cell Subpopulations and CLASI Score

We found a negative correlation between the disease activity and the percentage of Th22 cells in the epidermis and dermis (Spearman's rho: −0.464, *P* < 0.001; and 0.494, *P* < 0.001, resp.); similar findings were determined in circulating Th22 cell number (Spearman's rho: −0.355, *P* < 0.043).

A positive correlation was found between the CLASI activity score and circulating Treg cell percentage (Spearman's rho: +0.45, *P* = 0.009).

The other correlations were not significant.

### 3.9. Correlation among the Tissue and Circulating Cell Subpopulations

IL-22-producing cells found in tissue from cutaneous lupus patients had a positive correlation with the circulating CD4^+^/CD161^−^/IL-22^+^ T cell percentage (Spearman's rho: +0.665, *P* < 0.001). Analogous results were determined in regard to the cell percentage of Th1 (Spearman's rho: +0.436, *P* = 0.13) and pDCregs (Spearman's rho: +0.611, *P* < 0.001). The other correlations were not significant.

## 4. Discussion

Cutaneous lupus erythematosus is an autoimmune, chronic, and inflammatory disease with a broad range of cutaneous symptoms which include from a mild erythema to disseminated plaques, deformities and scars, photosensitivity, and oral ulcers. The pathogenesis of cutaneous lupus is multifactorial, heterogeneous, complex, and poorly understood [13].

Thus, we analyzed the presence of Th22 and IL-22 in blood and tissue samples from cutaneous lupus patients. IL-22 belongs to the IL-10 superfamily. It initiates innate immune response against pathogens in gut epithelial, skin, and respiratory cells and regulates tissue repair/regeneration and antibody production. Th22 subset differentiates from T naïve cells in response to TNF-*α* and IL-6. Th22 cells synthesize and secrete IL-26, IL-13, and IL-22; the former is the most relevant cytokine due to the fact that it plays an important role in cell proliferation and survival, antimicrobial peptide production, epithelial renewal, and immunity [14]. Nonetheless, information that is available regarding this cytokine in cutaneous lupus is scarce and controversial. Abnormal Th22 cell number and IL-22 expression have been determined in peripheral blood from patients with psoriasis, systemic sclerosis, and rheumatoid arthritis [15, 16].

In our patients with cutaneous lupus, we found that the Th22 percentage cell was significantly higher in the skin from DLE and in lesser extent in SCLE patients in comparison to healthy tissue. These findings had a positive correlation with those obtain by flow cytometry in circulating IL-22-producing CD4 T cells. In addition, a negative correlation between tissue and peripheral Th22 cell percentage with the CLASI score was found, meaning that higher activity scores imply lower percentages of Th22 cells. Based on these findings, IL-22 in cutaneous lupus might be considered as a cytokine that participates in tissue repair more than in inflammation.

Moreover, our data are in accordance with those obtained by Yang et al., regarding the increased number of Th22 cells in periphery. Similar to our data, Yang et al. did not find difference in Th22 cell frequency and IL-22 levels among DLE and SLCE patients. In contrast with our data, they observed a positive correlation between IL-22 serum levels and Th22 cell number [17].

Finally, IL-22 serum levels were lower in cutaneous lupus patients compared with healthy individuals and they did not have any correlation with the percentage of tissue and/or circulating Th22 cells. This suggests that IL-22-producing cell subsets secrete the cytokine locally but not systemically.

Similarly in patients with SLE, two studies have reported reduced levels of IL-22 [15, 18]. However the unexpected decrease of IL-22 in SLE patients remains controversial. Indeed some authors have suggested that the reduced serum levels may have an inverse correlation with the use of hormones and dexamethasone. Conversely other authors have described increased levels but only among patients with lupus nephritis. Thus it is clear that IL-22 may be involved in the pathogenesis of SLE and deserves further research [19].

On the other hand, Th17 cells produce IL-17 that stimulates T cells, increases the production of autoantibodies, inflammatory cytokines (TNF-*α*, IL-1*β*, IL-6, IL-8, IL-17, IL-22, etc.), and chemokines (CCL2, CCL7, CCL20, CXCL1, and CXCL5) [20, 21], and induces neutrophil recruitment through chemokine regulation [22]. As it was previously reported by Tanasescu et al. and Mikita et al., we determined a higher Th17 cell frequency in the tissue from DLE and in lesser extent in SCLE patients when compared to healthy skin and HSc. A similar profile was observed in circulating IL-17-producing CD4 T cells. Serum IL-17 levels were significantly higher in the DLE and SCLE patients compared with healthy subjects [23, 24]. Nonetheless, in contrast to Tanasescu et al. findings we determine more severe inflammatory response in DLE versus SCLE patients. These results and two case reports where treatment of cutaneous lupus with Ustekinumab improved cutaneous lesions indicate a potential role for IL-17 on the development of inflammation [25, 26].

Regarding IFN-*γ*, it is involved in the regulation of the immune and inflammatory responses. IFN-*γ* is produced by activated T cells and NKs. It potentiates the effects of the type I IFNs, recruits leukocytes to the infected tissue, resulting in increased inflammation, and stimulates macrophages to kill bacteria that have been engulfed. IFN-*γ* released by Th1 cells is also important in regulating the Th2 response. As IFN-*γ* is vitally implicated in the regulation of immune response, its production can lead to autoimmune diseases. It has been demonstrated that SLE patients have higher levels of this cytokine and they correlate with disease activity [27–29]. Moreover, IFN-*γ* inhibits collagen synthesis and induces the production of CXCR3, CXCL9, CXCL10, and CXCL11 in skin from patients exposed to UV radiation [30]. Furthermore, extraction of T cells from the skin of DLE patients showed a large number of IFN-*γ*-expressing cells [31]. However, its role in the pathogenesis of cutaneous lupus is still being determined. According to the limited literature, we also determined an increase in tissue and peripheral blood of IFN-*γ*-producing CD4 T cells in DLE and SCLE patients.

IL-4 is an anti-inflammatory cytokine that inhibits the synthesis of IL-1*β*, TNF-*α*, IL-6, IL-17A, and so forth. It regulates B cell proliferation and differentiation, and it is also a potent inhibitor of apoptosis. IL-4 is synthesized primarily by Th2 and it is required for the initiation and maintenance of fibrosis. IL-4-expressing CD4 Th2 subset is defined by the production of IL-4, IL-5, IL-9, and IL-13. Type 2 immunity suppresses the autoimmune disease mediated by Th1 cells [32], neutralizes toxins, maintains the metabolic homeostasis, regulates wound healing and tissue regeneration, and induces fibrosis enhancing collagen production and deposition [33]. IL-4-producing CD4 T cells in the skin and peripheral blood from our patients were higher versus healthy donors. Nonetheless, there was no difference among the cutaneous lupus subtypes. These data suggest that IL-4 could participate in wound healing and fibrosis and also in the suppression Th1 effector cells.

The term “regulatory cells” includes a variety of cell subpopulations specialized to exert cell extrinsic immunosuppression. Tregs modulate the natural course of protective immune responses in order to limit tissue damage and autoimmunity [34, 35]. Treg cells suppress immunologic responses in several steps: producing granzymes and perforins, by IL-2 depletion, secretion of suppressor molecules as IL-19 and TGF-*β*, and diminishing the functions of antigen presenting cells (APC) that can promote anergy or apoptosis of effector T cells. Several groups have analyzed the number of Treg cells in SLE patients and they have described a low cell number in active patients [36–39]. In a previous study, a low frequency of this population in skin lesions from patients with cutaneous lupus versus healthy controls was reported. Nevertheless there was no difference amongst SCLE and tumid lupus. In the present study, we documented an increase of skin from DLE and SCLE patients compared with healthy skin. In addition to the findings of Franz et al., Treg cell percentage was lower in both subgroups of cutaneous lupus in comparison to the HSc patients [34]. It is important to consider that numerical, phenotypic, and functional defects affect a range of Treg subsets. Thus, the increase of Treg cells in skin from our patients with cutaneous lupus could be related to the determination of a subpopulation of Treg cells (CD25^+^/Foxp3^+^) while, in most of the studies, immunohistochemistry has been done identifying only a single marker, Foxp3. It is well known that Foxp3 is also expressed in CD8^+^/CD25^+^ Tregs, CD8^+^/CD28^−^ Tregs [40], CD4^+^/IL-17^+^/Foxp3^+^ T cells [41], and CD19^+^/Foxp3^+^ B cells [42].

In addition to Tregs, a newly described population of regulatory B cells also contributes to immunosuppression both directly and via enhancement of Treg function. It is a CD19^+^CD24^hi^CD38^hi^ immature/transitional B cell subset that suppresses the differentiation of Th1 cells in an IL-10-dependent manner. It has been shown that, in SLE patients, the CD19^+^CD24^hi^CD38^hi^ B subset produces less IL-10 in response to CD40 stimulation and is unable to inhibit Th responses, suggesting that altered cellular function of the subpopulation in SLE may impact the immune effector responses in this autoimmune disease [43]. In the present study, we found a higher frequency of circulating CD19^+^/CD24^hi^/CD38^hi^/IL-10^+^ B cells in DLE patients versus healthy donors and SCLE patients.

Lastly, it is known that dendritic plasmacytoid regulatory cells (pDCregs) are a subpopulation expressing indoleamine 2,3-dioxygenase (IDO), an enzyme responsible of tryptophan metabolism that suppresses T effector cells activity and induces CD4^+^/CD25^hi^ regulatory T cells polarization* in vivo*. Deprivation of tryptophan by IDO halts the proliferation of T cells at mid-G1 phase, which in concert with the proapoptotic activity of kynurenine leads to developing immune tolerance. IDO has a selective and potential role for Th2 differentiation and is regulated positively during antigenic presentation and the union of CTLA-4/B7·1/B7·2 in lymphocytes and dendritic cells; in response to infection and circulation nucleic acid through TLR4 and TLR9 activation; and by tissue inflammation [44] and they are critical regulator of adaptive immunity contributing to inflammatory process.

As previously mentioned, we identified that both the inflammatory and anti-inflammatory response prevailed in DLE when compared with SCLE. In this sense, clinically SCLE is a photosensitive, nonfixed, nonscarring lesion whereas DLE have a tendency for scarring and atrophy [45].

A limitation of our study is that most of the patients were under a standard lupus treatment based on glucocorticoids (GCs) and immunosuppressive therapy at the time of the evaluation. GCs are still the angular stone of the treatment and they have a direct action on the Th1 cell polarization, which it turn modifies the balance and cytokine profile Th1/Th2 [46]. On the other hand, hydroxychloroquine has proved to inhibit the proinflammatory cytokine production by macrophages and the antigen presentation [47]. In this scenario, the differences in the cytokine levels and frequency of analyzed cell subsets in our study regarding to the previous findings could be due to the treatment.

Summing up, the present study shows an increment in the percentage of Th22, Th17, and Th1 cells as well as pDCreg cells in DLE patients versus SCLE. Our results suggest that cutaneous lupus is a disease that elapses with local and systemic inflammation, more intense in DLE compared with SCLE. The imbalance of pro- and anti-inflammatory cell subsets and cytokines favors the former, with the failure of the last one to maintain the homeostasis. Our results shed further light regarding the identification of diverse cell subpopulations and cytokines in the pathophysiology of DLE and SCLE. These findings certainly deserve to be studied in depth in order to evaluate the clinical relevance of these findings.

## Figures and Tables

**Table 1:** Demographic and clinical characteristics from patients with cutaneous lupus erythematosus.

	HD	HSc	DLE	SCLE	*P* value
	(*n* = 16)	(*n* = 5)	(*n* = 20)	(*n* = 15)	
*Demographics*					
Age (years)					
Median	45	29	39	35	
Range	(21–76)	(24–33)	(18–74)	(20–62)	**0.003**
Sex (female %)	100	100	95	93	NS
Disease duration (years)					
Median	—	—	7	6	NS
Range			(1–26)	(1–41)	

*Laboratory variables*					
Leukocytes (cells/mL)					
Median	5500	ND	5200	6100	NS
Range	(4200–8200)		(2400–6700)	(2200–15700)	
Lymphocytes (%)					
Median	29	ND	20	19	**0.028**
Range	(18–47)		(14–33)	(8–33)	
ESR (mmHg)					
Median	6	1	8	10	**0.0024**
Range	(2–18)	(1–15)	(2–28)	(2–48)	
Anti-dsDNA (IU/mL)					
Median			16	45	
Range	—	—	(8–111)	(12–447)	**<0.001**
C3 (mg/dL)					
Median			79	79	
Range	—	—	(24–134)	(29–130)	NS
C4 (mg/dL)					
Median	—	—	14	14	NS
Range			(8–29)	(6–37)	

*Clinical variables*					
CLASI activity score					
Median			11	21	
Range	—	—	(2–25)	(9–37)	**<0.001**
Prednisone (mg/day)					
Median			5	10	
Range	—	—	(0–30)	(0–50)	**0.0037**
Antimalarial (%)	—	—	75	67	**0.043**

HD: healthy donors; HSc: hypertrophic scar; DLE: discoid lupus erythematosus; SCLE: subacute cutaneous lupus erythematosus.

**Table 2:** Cytokine expression and regulatory cells in tissue from patients with cutaneous lupus erythematosus.

	Epidermis				Dermis			
	HD (*n* = 16)	HSc (*n* = 5)	DLE (*n* = 20)	SCLE (*n* = 15)	HD (*n* = 16)	HSc (*n* = 5)	DLE (*n* = 20)	SCLE (*n* = 15)
IL-22-expressing cells (%)								
Mean ± SEM	5.7 ± 0.7	15.6 ± 0.5^a^	23.4 ± 2.1^b,d^	16.1 ± 1.8^c,f^	4.7 ± 1.0	17.3 ± 0.8^a^	28.9 ± 2.5^b,d^	17.0 ± 1.9^c,f^
Median	5.0	16.0	21.0	16.0	3.0	17.5	28.0	19.0
Range	2.0–10.5	14.0–18.0	7.0–49.0	2.0–5.5	1.0–14.0	15.0–22.0	8.0–55.0	7.0–31.0

IL-17A-expressing CD4^+^ cells (%)								
Mean ± SEM	2.8 ± 0.5	5.0 ± 0.7	27.1 ± 1.8^b,d^	11.9 ± 1.5^c,e,f^	2.1 ± 0.5	4.1 ± 0.5	29.5 ± 1.8^b,d^	12.4 ± 1.1^c,e,f^
Median	2.0	5.0	24.0	13.0	2.0	4.5	28.5	13.5
Range	1.0–8.0	2.0–8.0	19.0–44.0	3.0–21.0	0.0–7.0	1.0–6.0	20.0–50.0	4.0–18.0

IL-4-expressing CD4^+^ cells (%)								
Mean ± SEM	3.0 ± 0.9	8.5 ± 0.9^a^	12.6 ± 1.4^b^	11.0 ± 1.1^c^	3.2 ± 0.9	13.8 ± 0.5^a^	12.3 ± 1.3^b^	11.8 ± 1.5^c^
Median	2.0	9.0	12.0	10.5	2.5	14.0	13.0	11.0
Range	0.5–15.0	3.0–14.0	4.0–25.0	5.5–18.0	0.0–13.0	12.0–17.0	1.5–21.0	4.0–22.0

IFN-*γ*-expressing CD4^+^ cells (%)								
Mean ± SEM	2.2 ± 0.5	7.5 ± 0.8^a^	11.0 ± 0.8^b,d^	8.0 ± 1.4^c,f^	2.5 ± 0.7	9.0 ± 0.5^a^	11.3 ± 0.7^b,d^	7.7 ± 1.1^c,f^
Median	2.0	8.0	10.0	6.0	2.0	9.0	11.0	6.0
Range	0.0–6.0	2.0–11.0	4.5–18.0	2.5–22.0	0.0–9.0	7.0–12.0	0.5–18.0	3.5–16.0

Foxp3-expressing CD25^+^ cells (%)								
Mean ± SEM	5.4 ± 1.0	45.9 ± 1.1^a^	14.8 ± 1.1^b,d^	12.5 ± 1.2^c,e^	5.4 ± 1.2	55.1 ± 0.8^a^	17.8 ± 1.1^b,d^	15.0 ± 1.1^c,e^
Median	5.0	45.5	16.0	12.0	3.0	56.0	17.5	15.0
Range	1.0–14.0	40.0–52.0	3.5–23.0	7.0–24.0	0.5–15.0	50.0–58.0	11.0–27.0	9.0–22.0

IL-10-expressing CD20^+^ cells (%)								
Mean ± SEM	7.3 ± 1.2	11.4 ± 0.6	10.9 ± 0.8	8.9 ± 1.4	8.8 ± 1.5	16.7 ± 0.8^a^	12.9 ± 1.2	12.3 ± 1.3
Median	5.5	10.5	10.5	8.0	6.5	16.0	13.0	11.3
Range	2.0–15.0	9.0–14.0	3.0–18.0	1.0–15.5	2.0–20.0	13.0–22.0	1.5–23.0	6.0–24.0

IDO-expressing CD123^+^ cells (%)								
Mean ± SEM	4.0 ± 0.7	17.1 ± 1.6^a^	9.9 ± 0.9^b,d^	5.0 ± 1.0^e,f^	4.0± 0.5	20.7 ± 1.2^a^	12.7 ± 1.2^b,d^	8.2 ± 0.9^c,e,f^
Median	4.0	16.0	8.5	4.0	4.0	20.0	11.0	8.0
Range	1.0–11.0	12.0–26.0	4.0–18.0	1.0–12.0	1.0–8.0	16.0–26.0	4.5–29.0	4.0–16.0

HD: healthy donors; HSc: hypertrophic scar; DLE: discoid lupus erythematosus; SCLE: subacute cutaneous lupus erythematosus. ^a^HD versus HSc: *P* < 0.05; ^b^HD versus DLE: *P* < 0.05; ^c^HD versus SCLE: *P* < 0.05; ^d^HSc versus DLE: *P* < 0.05; ^e^HSc versus SCLE: *P* < 0.05; ^f^DLE versus SCLE: *P* < 0.05.

**Table 3:** Cytokine expression and regulatory circulating cells in patients with lupus erythematosus.

	HD (*n* = 16)	HSc (*n* = 5)	DLE (*n* = 20)	SCLE (*n* = 15)
CD3^+^/CD4^+^/CD161^−^/IL-22^+^ cells (%)				
Mean ± SEM	2.4 ± 0.1	6.0 ± 0.8^a^	12.4 ± 0.4^b,d^	10.5 ± 0.3^c,e,f^
Median	2.8	5.9	12.1	10.3
Range	1.5–3.0	4.4–8.9	10.2–15.2	9.5–12.5

CD3^+^/CD4^+^/CD161^+^/IL-17A^+^ cells (%)				
Mean ± SEM	2.1 ± 0.1	6.0 ± 0.4^a^	10.3 ± 0.3^b,d^	5.2 ± 0.4^c,f^
Median	2.2	6.4	10.3	4.7
Range	1.1–2.8	4.3–6.7	8.5–11.9	3.9–9.0

CD3^+^/CD4^+^/CD14^−^/IL-4^+^ cells (%)				
Mean ± SEM	2.0 ± 0.1	8.4 ± 0.6^a^	6.6 ± 0.3^b,d^	7.1 ± 0.2^c,e^
Median	2.1	8.6	6.9	7.3
Range	1.2–2.9	6.2–9.6	4.6–8.8	5.5–8.4

CD3^+^/CD4^+^/CD14^−^/IFN-*γ* ^+^ cells (%)				
Mean ± SEM	1.9 ± 0.2	5.6 ± 0.6^a^	9.0 ± 0.2^b,d^	7.0 ± 0.2^c,e,f^
Median	1.7	6.1	8.7	6.8
Range	1.1–3.0	3.2–6.8	8.0–10.9	6.1–8.4

CD3^+^/CD4^+^/CD25^hi^/Foxp3^+^ cells (%)				
Mean ± SEM	8.4 ± 0.2	10.1 ± 0.3^a^	8.1 ± 0.2^d^	8.3 ± 0.3^e^
Median	8.5	10.4	8.3	8.5
Range	7.1–9.2	9.5–10.7	5.5–9.3	6.6–9.8

CD19^+^/CD24^hi^/CD38^hi^/IL-10^+^ cells (%)				
Mean ± SEM	10.3 ± 0.3	12.1 ± 1.1^a^	12.9 ± 0.4^b^	10.8 ± 0.4^f^
Median	10.0	10.8	13.0	10.3
Range	7.8–12.5	9.8–15.5	10.4–14.8	9.1–13.0

CD123^hi^/CD196^+^/IDO^+^ cells (%)				
Mean ± SEM	18.6 ± 0.4	19.0 ± 0.9	33.2 ± 0.7^b,d^	23.4 ± 0.7^c,e,f^
Median	18.4	18.6	32.3	22.6
Range	15.2–22.4	17.0–21.9	28.5–39.0	20.0–27.1

HD: healthy donors; HSc: hypertrophic scar; DLE: discoid lupus erythematosus; SCLE: subacute cutaneous lupus erythematosus. ^a^HD versus HSc: *P* < 0.05; ^b^HD versus DLE: *P* < 0.05; ^c^HD versus SCLE: *P* < 0.05; ^d^HSc versus DLE: *P* < 0.05; ^e^HSc versus SCLE: *P* < 0.05; ^f^DLE versus SCLE: *P* < 0.05.

**Table 4:** Cytokine levels in serum from patients with lupus erythematosus.

	HD (*n* = 16)	HSc (*n* = 5)	DLE (*n* = 20)	SCLE (*n* = 15)
IL-22 (pg/mL)				
Mean ± SEM	229.0 ± 28.1	38.5 ± 2.2^a^	211.8 ± 53.1^d^	96.6 ± 16.3^c,f^
Median	211.2	37.5	126.4	101.9
Range	106.3–517.5	32.2–45.2	85.8–955.5	34.4–249.9

IL-17A (pg/mL)				
Mean ± SEM	2.2 ± 0.2	6.5 ± 1.8^a^	5.8 ± 0.4^b^	4.5 ± 0.4^c,f^
Median	2.0	8.1	5.3	4.5
Range	1.1–3.9	0.9–10.0	3.4–10.4	2.3–6.8

IL-10 (pg/mL)				
Mean ± SEM	2.5 ± 0.3	2.3 ± 0.4	7.1 ± 2.6	2.5 ± 0.2^f^
Median	2.1	2.1	3.2	2.5
Range	1.6–5.4	1.6–3.5	2.0–51.9	1.1–3.5

HD: healthy donors; HSc: hypertrophic scar; DLE: discoid lupus erythematosus; SCLE: subacute cutaneous lupus erythematosus. ^a^HD versus HSc: *P* < 0.05; ^b^HD versus DLE: *P* < 0.05; ^c^HD versus SCLE: *P* < 0.05; ^d^HSc versus DLE: *P* < 0.05; ^e^HSc versus SCLE: *P* < 0.05; ^f^DLE versus SCLE: *P* < 0.05.

## Abbreviations

ACLE: Acute cutaneous lupus erythematosus
AP: Alkaline phosphatase
APC: Allophycocyanin
Bregs: Regulatory B cells
CD: Cluster differentiation
CCLE: Chronic cutaneous lupus erythematosus
CLASI: Cutaneous Lupus Erythematosus Disease Area and Severity Index
DAB: Diaminobenzidine
DLE: Discoid lupus erythematosus
ELISA: Enzyme-linked immunosorbent assays
ESR: Erythrocyte sedimentation rate
FITC: Fluorescein isothiocyanate
FMO: Fluorescence minus one
Foxp3: Forkhead box P3
HD: Healthy donors
HRP: Horseradish peroxidase
IDO: Indolamine 2,3-dioxygenase
IFN-*γ*: Interferon-gamma
IL: Interleukin
LINK: Dextran polymer coupled with secondary antibodies against mouse and rabbit immunoglobulins
PBMCs: Peripheral blood mononuclear cells
pDCs: Plasmacytoid dendritic cells
PE: Phycoerythrin
PeCy5: Phycoerythrin Cychrome 5
SCLE: Subacute cutaneous lupus erythematosus
SD: Standard deviation
SEM: Standard error of the mean
SLE: Systemic lupus erythematosus
Th: T helper cells
Tregs: Regulatory T cells.
